# Bibliographical Notices

**Published:** 1846-06

**Authors:** 


### BIBLIOGRAPHICAL NOTICES.

*A Treatise on Mediate Auscultation, and on Diseases of the Lungs and Heart.* By R. T. H. Laennec, Professor of the College of France, and to the Faculty of Medicine of Paris, &c. &c. *With the notes and additions of* M. Mer. Laennec, D. M. P., and of M. Andral, Professor to the Faculty of Medicine of Paris, Consulting Physician to the King, &c. *Translated from the latest edition by a Member of the College of Physicians.* Edited by Theophilus Herbert, M. D. *With practical notes, condensed from the Lectures of* F. H. Ramadge, M. D., Oxon. Fellow of the Royal College of Physicians, Senior Physician to the Infirmary for Asthma, Consumption, and other Diseases of the Lungs, &c. *With plates.* 8vo. pp. 862. Lond., 1846.

We have copied the *lengthy* and ostentatious title page of this *phenomenon*,—for such the present edition of Laënnec must be regarded,—if by phenomenon we mean any thing extraordinary, but not if we regard the word to signify something unexpected, inasmuch as there is no form which empiricism and adulation may not assume, and none, therefore, that ought properly to excite surprise. The name of Dr. Ramadge has been long before the public rather than the profession, as that of one respectable by education, and, we may say, in one sense, by position, who has descended to a level with the veriest empirical pretender as a professed "consumption curer;" and whose views and agents have met with but little respect from the regular faculty; yet who has not wanted followers to trumpet his fame, and to obtain pelf and notoriety from their overdrawn mercenary eulogies.

The work before us is—we had almost said—the *most* marked example of unworthy Boswellism we have ever met with. It has had, however, rivals on this side of the Atlantic; but we hope and trust to witness no more of them. Johnson was illustrated by his garrulous biographer; and we rise from the perusal of his Life by Boswell with still greater admiration for the hero of the work, but with feelings akin to contempt for the biographer. Would that we could say, that in more recent cases, either the sketcher or the sketched was exalted in our estimation. But let us hear Dr. Theophilus Herbert in the dedication to his preceptor—and doubtless his patron—Dr. Ramadge.

"Trusting that the manner in which my Editorial task has been performed will meet your approbation, and be found not unworthy of your high name, I now deliver this book into your hands. Should any inaccuracies be found in it imputable to the Editor, (and for such only am I accountable,) they will, I am sure, be more than redeemed by the extensive, valuable, and practical annotations formerly derived from you, and added to the present edition of Laënnec. Your extensive experience in diseases of the chest—your having devoted the greater portion of your professional life to the investigation of thoracic pathology in particular,—your having been for more than a quarter of a century connected with the Infirmary for Asthma, Consumption, &c., where, exclusive of other sources, you have enjoyed the amplest opportunities for studying this very important class of diseases—all these circumstances point out Dr. Ramadge as the individual to whom Laënnec's great work should most appropriately be inscribed;—and if anything were wanted to strengthen your title to this, you have now attained the great and undisputed honor of having introduced into medical practice the most successful and rational mode of treating a disease, which has proved the scourge of the most intellectual [?] portion of the human race, and baffled the skill of the greatest physicians of ancient and modern times—I mean Pulmonary Consumption; a mode of treatment, moreover, not the result of mere hypothesis, but derived from an attentive observation and close imitation of nature herself. These reasons, and above all your having virtually made the present edition of Laënnec undeniably your own, by your extensive and valuable contributions to it, render it imperative on me, in point of justice, to dedicate the work to you."

This is a specimen of the "puff direct," not excelled by what we witness in the advertising corners of our daily papers, from the grateful afflicted, testifying to the value of a nostrum which they have been employing. The "puff collateral" consists in the association of the name of Ramadge with that of the illustrious Laënnec,—"*ego et rex meus;*" for so we doubt not Dr. Ramadge regards the association. What necessity was there, that the work of Laënnec, after having been translated, and edition after edition published by Dr. Forbes, should be again translated by another "Member of the College of Physicians," unless it were to announce to the *laity*, that Dr. Ramadge is worthy of being placed in the same elevated position as an explorer and treater of diseases of the chest as Laënnec.

But we doubt not, that most of our readers are as ignorant as ourselves of the peculiar views of Dr. Ramadge, which have excited such overflowing admiration in his editor. We shall therefore give them in the language of the latter.

"It struck Dr. Ramadge at the outset of his inquiries into this interesting subject, [the nature and treatment of pulmonary disease] that persons who, after evincing undoubted symptoms of pulmonary phthisis, subsequently became affected with emphysema, were rarely carried off by phthisis. Again, he observed, that individuals, formerly consumptive, and afterwards becoming subjects of chronic catarrh, seldom fell victims to phthisical disease. He also observed that individuals afflicted with disease of the heart were hardly ever attacked with phthisis, and therefore he very sedulously commenced inquiring what was the cause of this immunity from phthisis in those suffering from these different diseases—what was the common quality which they all possessed, and which proved so powerful an opponent to the attack of phthisis. The result of his inquiries was, that these affections induced a state of the pulmonary tissue, which counteracted the progress of tubercular deposition, and so checked the disease. To enter more into particulars, a person who has been for some time labouring under pulmonary phthisis contracts a catarrh; now one of the effects of this is to produce an intumescence of the bronchial mucous membrane; this intumescence retards the expiration of the air, the expirating power being so much weaker than the inspirating; the air becomes imprisoned in the air-cells, the lungs are exercised in consequence, and their volume is augmented; the effects of which will be: 1. To approximate the walls of any cavity that may exist at the time. 2. To prevent the further deposition of tuberculous matter, on the principle that the muscles of voluntary motion are seldom the seat of tubercles. To explain this point more fully, we remark, that as soon as phthisis commences, the chest generally becomes contracted. The trachea, by retaining its original size, is found comparatively too large for this diminished volume of the lung; the exit of the air is then too free to offer any antagonism to the farther contraction of the chest, and consequently to the advance of the disease: the result of which would be the deposition of fresh crops of tubercles, and accordingly their extensive diffusion throughout the lower lobes of the lungs. In a similar manner cardiac diseases may prove effectual antagonisms in pulmonary consumption. In all lesions of the heart there is to be found congestion of the venous system: there is also tumefaction of the bronchial mucous surface in consequence of the difficulty which the bronchial veins experience in transmitting their blood into the larger venous trunks destined to receive it. The effect of this is, that the air becomes imprisoned in the same manner as in the case of catarrh, and the lungs are thereby augmented in volume. The tendency of this enlargement of the lungs is to destroy the disposition to form secondary crops of tubercles, such enlargement being decidedly unfavorable to the continuance of the scrofulous diathesis. The experience of Dr Ramadge has led him to establish a regular connexion between tuberculous disease and scrofula in any part of the body. * * * * We have now seen how the existence of catarrh, disease of the heart, &c., have the power of staying the progress of pulmonary consumption. That these means of arrest, thus presented by mere accident, are attended with considerable inconvenience, no one will pretend to deny. Nay, more; it is well known that such diseases as those we have mentioned as capable of suspending phthisis, are frequently themselves sufficiently formidable to compromise life. Reflecting on the principle on which these affections acted in impeding the further contraction of the chest, and so preventing the further deposition of tubercles, the distinguished discoverer, Dr. Ramadge, very naturally proceeded to consider whether some mechanical expedient could not be devised, by which the operations of nature might be imitated, at the same time, that the disadvantages above alluded to might be eschewed. We have observed that the benefit derived from antagonistic diseases depended upon this circumstance, that they had the effect of retarding the expiratory process, and thereby increasing eventually the volume of the lungs. Now he very reasonably inquired whether such a result could not be brought about mechanically, and it was then the happy idea occurred to him of employing the inhaling tube. The principle on which it is constituted is, to impede or retard the expiratory process, and thereby to produce an increase in the volume of the lungs. In order to explain how impeding or retarding the expiratory process will have the effect of increasing the volume of the lungs, very few words will suffice. When the function of expiration is retarded by any cause, the obvious and immediate result will be that a portion of the air becomes imprisoned within the air-cells; this will bring about, as it were, an emphysematous state of the lungs, whereby their volume will be expanded to a certain degree, and the walls of the chest will be pushed out in every direction; this retardation of the expiration is effected by means of the valve placed at one extremity of the inhaling tube. Now for the application of this principle to the treatment of pulmonary consumption. In phthisis the lungs are not fully inflated, and the parietes of the chest undergo gradual contraction. The healthy relation between the powers of inspiration and expiration is lost. The great air-tube is proportionally too wide for the diminished volume of the lungs. Whatever restores the wholesome balance between the lungs and the windpipe, contributes to arrest the progress of tubercular disease. This restoration of the equilibrium between inspiration and expiration, which we have now seen to be possible through the agency of the tube, is occasionally accomplished, though imperfectly, by the supervention of some other affection, such as enlarged tonsils, catarrh, asthma, disease of the heart, &c." p. viii.

Such are the principles and practice, "not the result of mere hypothesis, but derived from an attentive observation and close imitation of nature herself," with which the profession are to be enlightened by this "high name," "distinguished discoverer," "high authority," &c. &c., for there is no end to the encomiums showered on Dr. Ramadge by his "grateful" and admiring pupil; and we doubt not, that multitudes of "facts," so called, exist to sustain him; yet the profession have been singularly ungrateful. They have treated him, indeed, with marked neglect; they have believed, that his "hypothesis"—for so we must term it—is unfounded, and his experience fallacious. Nay, more, they have been disposed to place him on the same level as the advertising consumption-curers of the day, but no higher,—if anything, indeed, somewhat lower, inasmuch as they had a right to expect better things from him. For our own part we do not know a respectable practitioner who has the slightest confidence in either his principles or his practice. We find, however, from the editor's statement, that the mechanical treatment, notwithstanding our want of information on the matter, is extensively used.

"The employment," he says, "of the mechanical apparatus in the treatment of all affections of the chest, is now widely spread over many parts of the continent of Europe, and has been introduced into the principal cities of the United States, through the agency and influence of several American physicians, who witnessed Dr. Ramadge's successful practice in pectoral disease. Among these he knows of none who have been more successful in introducing the use of mechanical respiration than his talented friend, Dr. J. M. Howe, of New York." p. 862.

But we have occupied too much space with Dr. Ramadge, and his enamoured disciple. Nothing but the association of his name with that of Laënnec would have induced us to take so much notice of him. Possessing—as we do—an excellent translation by a most eminent physician—himself distinguished for his acquaintance with the nature and treatment of thoracic diseases—we could not suffer to pass, without comment, the publication of another, where the apparent object was so little praiseworthy.

---

*The Dublin Quarterly Journal of Medical Science, (new series,) No.* 1, *February,* 1846. 8vo. pp. 276. Dublin: Hodges & Smith.

It is not our practice to notice periodicals at much length, or even to speak of their contents in detail, but the character of the present work, and the circumstances under which it appears, claim for it more than a common announcement. It is, we believe, the second attempt to establish a *quarterly* journal devoted to medical science in the Irish metropolis, and the only one, if we are not mistaken, which has emanated from thence for more than a quarter of a century. And yet, no place, in proportion to its population, has been more distinguished by the number and ability of its physicians, surgeons, and chemists, than the city of Dublin, nor have those of any other country contributed more to the advancement of science and the adornment of medical literature. Within the period mentioned, a number of medical journals, under various denominations,—weekly, monthly, and bi-monthly,—have started into existence, and for longer or shorter periods been sustained, and some, as the *Dublin Medical Press,* and the *Dublin Hospital Gazette,* continue to be the vehicles of valuable medical intelligence.

In the year 1832, the Dublin Journal of Medical and Chemical Science, was commenced and ably conducted by the eminent chemist, Dr. Kane. Two years after, Dr. Kane retired from the editor's chair, and was succeeded by Drs. Graves and Stokes, by whom it was conducted under the name of the *Dublin Medical*

*Journal*, until the year 1842, "when their increasing avocations obliged them to resign it into the hands of Messrs. Hamilton and MacDonnell." Now, instead of appearing monthly, it is to be issued quarterly, with a new title, and under the direction of a new board of editors; and, distinguished as it has heretofore been for ability, and the avoidance of every thing offensive to good taste, the character of those by whom it is hereafter to be conducted, no less than the evidence furnished in the number before us, afford a sure guarantee that it will maintain its position among the ablest and most valuable medical periodicals of the day.

In the general arrangement of its matter, nearly the same order is pursued as in the Examiner. It is divided into three parts—Part I. is assigned to Original Communications; Part II., Reviews and Bibliographical Notices; Part III., Reports, Retrospects, and Scientific Intelligence.

Preceding the first part, we have "the Editor's Preface," consisting chiefly of "the history of periodical medical literature in Ireland, including notices of the medical and philosophical societies of Dublin." No one can read this able article without feeling an echo in his own breast to the following eloquent and truly filial expression of the editor:

"No matter how the early political history of our native land may be darkened;—of her literary and scientific history, her monuments and her antiquities, we have all, as Irishmen, a just right to glow with honest pride."

The original articles are contributed by some of the first writers of the day—viz.: Crampton, Graves, Halpin, and Neligan, and are worthy of their source. That by Sir P. Crampton, is a lecture on lithotrity, and is a most instructive and candid examination of the mooted points of the subject.

The second article is by Dr. Graves, "on the Law which regulates the Relapse-Periods of Ague." Dr. Graves, "having noted, with much anxiety and accuracy, the course of a quartan ague for twenty-seven months," has "constructed a table for the purpose of obtaining a connected view of the number and dates of the fits." This table, he thinks, contains "*data* which authorize us in concluding that the law regulating the periodicity of agues, applies not only to the succession of paroxysms, but is extended to the free intervals between them—in other words, that the same law of periodicity which governs the disease while it occasions fits, continues likewise to preside over its latent movements during the interval when no fit occurs, and thus the true periodic rate is carried on, though, as in a clock from which the striking weight has been removed, the usual signal does not mark the termination of each certain definite portion of time."

"The law, now for the first time brought to light," he remarks, "exhibits a new example of the tenacity with which periodicity clings to a disease, when once firmly impressed on it, and recalls to mind a very similar phenomenon observed with respect to the catamenia, which, having been suppressed many months, not unfrequently re-appear on the very day on which the monthly period would have occurred, had no such suppression taken place."

It seems to us rather too hasty to predicate a *law* on the observations afforded by a single case, however numerous those observations, as they certainly were in the instance adduced by Dr. Graves; but the hint thrown out by this eminent observer will doubtless lead to a closer attention to the phenomena presented in the progress of periodical diseases, and especially on the point alluded to, and if the view presented by him shall be sustained, it will be a great point gained in pathological science, and must lead to a better and more successful treatment of a perplexing class of diseases.

Dr. Graves thinks the facts which he has observed show "that ague is at first purely periodic, the health being totally unaffected during the interval between the attacks, but as the disease becomes rooted in the constitution, the intervals become less purely healthy"—which is merely affirming that the continuance of the disease produces other and more positive lesions. Dr. G. is consequently an advocate for an early and prompt interruption of an ague, by the use of full doses of quinine. He differs, however, from those who think it expedient to continue its employment after the arrest of the paroxysms, or that it is necessary to leave it off gradually. "The quinine," he remarks, "is the proper antagonist of the fit, and while the fits require this medicine, it is borne well by the constitution. On the contrary, when the fits are absent, its curative effects appear to be diminished, and the constitution becomes so accustomed to it, that, when the disease again requires it, the medicine no longer exerts its anti-aguish influence." Our limits preclude a further notice of this curious and suggestive paper.

Part II, devoted to Reviews and Bibliographical Notices, occupies more than a hundred pages. Most of the works reviewed, have already been noticed in the Examiner, and need not be specified. This department of our new cotemporary, if so we may call it, is sustained with great ability. Some of the authors whose works are examined, will doubtless writhe under the severe castigation which is inflicted; but even they will be consoled by the reflection that there is nothing brutal in the process, for every joint that is separated and every cavity that is exposed, is done in the gentiest manner and with the most delicate instruments. One article in this *part* has particularly struck us as marked by the greatest vigour of argument and aptness of analysis—it is on *Homœopathy and Homœopathic writings.* Any man who can read it and continue to listen to the jargon, must be as blind to reason as are the eyeless fishes of the caves to the rays of the sun.

---

Sydenham Society's Works. *Animal Chemistry with reference to the Physiology and Pathology of Man.* By Dr. J. Franz Simon, Fellow of the Society for the advancement of Physiological Chemistry at Berlin, &c. &c. *Translated and Edited by* George E. Day, M. A. and L. M., Cantab., Licentiate of the Royal College of Physicians. Vol. 2, pp. 560, and two plates. London, 1846.

This beautiful volume is the concluding portion of M. Simon's excellent work on Animal Chemistry; and the first of the publications of the Sydenham Society for the year 1845. In regard to the value of this selection by the Society we apprehend there can be but little difference of opinion amongst the members. By those who look only for the directly practical, it may, doubtless, be regarded as too purely scientific; but their number is gradually becoming more and more restricted; and few, at the present day, would be hardy enough to pronounce any authoritative work on organic chemistry useless or unnecessary. We notice in some of the British journals objections to Paulus Ægineta, but they appear to us to rest upon little true foundation. It is most desirable to possess in epitome the sentiments of the ancients on medical topics;—and by means of the text of Paulus, and the learned commentaries of Mr. Adams, this is most satisfactorily attained in the Sydenham edition. The work of least general utility, it appears to us, is the one consisting of Tracts on Aneurism, which was issued last year. All tastes must, however, be suited; and we know how impossible it is, in any such undertaking, to give universal, and how difficult to give even general, satisfaction.

The present volume contains ten chapters, from 3 to 13 inclusive; treating respectively of—The secretions of the Chylopoietic viscera and the Theory of Digestion,—Milk,—Secretions of the Mucous Membrane—Secretions of the External Skin,—The Urine,—The Secretions of the Lachrymal, Meibomean and Ceruminous glands,—Secretions and Fluids of the Generative organs,—The Intestinal Secretions,—The component parts of the animal body,—Solid morbid products,—and fluid products of disease. At the end of the volume are two Appendixes treating of subjects not previously noticed, or to which it was necessary to make additions.

It is impossible to analyze a work of this kind, and unnecessary even to speak of it in general terms after the notice which we gave of the first volume. The chapter on the urine is exceedingly full, and we are pleased to see, that due attention has been paid by M. Simon to the excellent paper on Kiestein, or, as M. Simon calls it, *Kystein*, published a few years ago by Dr. E. K. Kane, of this city, now of the United States Army. From the results of his observations M. Simon concludes, "that kystein is not a new and distinct substance, but a protein compound, whose function is undoubtedly and closely connected with the lacteal secretion." And he adds: "From the observations of Kane and myself, it seems to follow that pregnancy may exist without the occurrence of kystein in the urine; if, however, there is a probability or possibility of pregnancy, and kystein be found in the urine, then the probability is reduced almost to a certainty. We are unable to draw any positive inferences respecting the stage of pregnancy from the appearance of kystein." p. 331.

The additions to the volume by Dr. Day are numerous and important; and the whole work is *à portée* with the existing condition of animal chemistry.

This single volume—we may add—is worth the whole year's subscription to the Society—*five dollars;*—and yet two more volumes are required to make the complement for the year. One of these has appeared in England;—"Hasse's Pathological Anatomy"—and will soon be in the hands of the subscribers in this country. The third—the concluding volume of Paulus of Ægina—will be issued soon.

---

*Elements of Physiology, including Physiological Anatomy, for the use of the Medical Student.* By WILLIAM B. CARPENTER, M. D., F. R. S., etc. etc. *With one hundred and eighty Illustrations.* 8vo. pp. 566. Lea & Blanchard. Philadelphia, 1846.

This is a new work by Dr. Carpenter, the sheets of which were received as they issued from the London press, and immediately reprinted, by the eminent publishers to whom we are indebted for the copy before us. By this arrangement, the work is published simultaneously, or nearly so, both in London and Philadelphia.

"The present volume owes its origin to a desire," says the author, "on the part of the Publisher, that an elementary treatise on Physiology should be added to the series of admirable Student's Manuals, on the various departments of Medical Science, which he has already issued."

The American Publishers have substituted the word "*Elements*" for "*Manual*," as written by the author, on the title page. The change, we suppose, makes the title more imposing, and as the work is truly *elementary* in its scope and character, the license would be allowable enough if it were not that the author, who certainly has the best right to christen his own progeny, had called it by another name. On this point we may be too sensitive, but we really have a great objection to alterations in the title or text of a book by any one but the author himself.

In the preparation of the work, the object of the author has been "to convey to the Student as clear an idea as possible of the principles of the science, to point out the manner in which these principles should be applied, and to give an outline of the most important facts which indicate the nature of the various changes taking place in the living organism. In following out this intention he has thought it right to adopt a plan which, so far as he knows, is a novel one:—namely, to commence his exposition of the characters of Organized Structures and of Vital Phenomena by a full account of the Development and Metamorphoses of *Cells,* and of the purposes which these effect in the living body, either in their original or in their altered condition. He is of opinion that the inferences which may be drawn from the observations on this subject, that have rapidly accumulated during the last few years, are entitled to hold the same rank in Physiological Science as that taken by the doctrines of Mutual Attraction in General Physics, or of Elective Affinity in Chemistry; and that the enunciation and development of these should consequently hold the first place in an Elementary Treatise on Physiology."

Those who have occasion for an Elementary Treatise on Physiology, cannot do better than to possess themselves of the Manual of Dr. Carpenter; such, however, as desire a fuller and more comprehensive account of the science, will of course consult the ampler works of the author, of Müller, or Dunglison. The illustrations in the present volume are exceedingly creditable.

---

*New Elements of Operative Surgery.* By ALFRED A. L. M. VELPEAU, etc. etc. *Translated by* P. S. TOWNSEND, M. D., etc. *Under the supervision of, and with notes and observations by,* VALENTINE MOTT, M. D., etc. etc. etc. Vol. 2. 8vo. pp. 992. J. & H. G. Langley, New York: 1846.

We cannot too strongly commend the industry of Dr. Townsend, in so rapidly bringing out this large and important publication. The third volume, which is to complete the work, we are told will be put to press in July next, and if the health of Dr. Mott and of the Translator and Editor be spared, will doubtless be urged forward with the expedition which has marked the progress of the two ponderous volumes now before us.

The Operative Surgery of Velpeau is conceded on all hands to be a work of vast labour and research, and in the highest degree creditable to the author; and the numerous and valuable additions by Dr. Mott, which constitute a very large amount of the present publication, render it still more complete as a book of reference on most, if not all, of the subjects demanding operations,—both as to the propriety of resorting to the knife, and the various and most approved methods of proceeding.

The subjects comprised in volume second are: Lesions of arteries and their treatment; of the venous system; of the lymphatic system; of the nervous system; amputations; exsection or excision of bones; exsection or excision of the articulations; trephining; tumors, &c. These various subjects are treated of at great length, particularly operations on the large arteries and excisions of bones, in which Dr. Mott's operations, their importance, originality, mode, &c., are particularly dwelt upon.

When we announced the appearance of the present volume a short time since, it was our intention, ere this, to have given a more particular notice of its contents, but the near approach to completion of the publication induces us to withhold what we may find occasion to say on the various subjects of chief importance of which it treats, until the whole is before us. By this course we shall anticipate nothing, and be the better prepared to do justice from a view of the whole, without prejudice from impressions derived from the examination of isolated portions.

---

*Twenty-Ninth Annual Report on the State of the Asylum for the relief of persons deprived of the use of their reason. To which is added an account of the Asylum. Published by direction of the Contributors.* Philadelphia, 1846.

This is a private institution, owned and controlled by members of the Society of Friends belonging to the Philadelphia Yearly Meeting. Under the care of its skilful Physicians and the vigilance of the Board of Managers, it continues to realize the benevolent anticipations of its founders.

We learn from the Report that the number of patients at the beginning of the year was— - - - - - 58

Received during the year, - - - - - 26

Discharged or died, - - - - - - 34

Remaining in the Asylum 3d mo. (March) 1st, 1846,—50

Of the thirty-four patients discharged there were—

| | | |
|---|---|---|
| Restored, | 15 | |
| Improved, | 6 | |
| Stationary, | 7 | |
| Died, | 6 | —34 |

Of those remaining there are—

| | | |
|---|---|---|
| Restored, | 4 | |
| Convalescent, | 2 | |
| Improved, | 4 | |
| Unimproved, | 40 | —50 |

